# Functional Epithelium Remodeling in Response to Applied Stress under *In Vitro* Conditions

**DOI:** 10.1155/2019/4892709

**Published:** 2019-05-19

**Authors:** Ivana Pajic-Lijakovic, Milan Milivojevic

**Affiliations:** Faculty of Technology and Metallurgy, University of Belgrade, Karnegijeva 4, Belgrade, Serbia

## Abstract

Mathematical modeling is often used in tissue engineering in order to overcome one of its major challenges: transformation of complex biological and rheological behaviors of cells and tissue in a mathematically predictive and physically manipulative engineering process. The successive accomplishment of this task will greatly help in quantifying and optimizing clinical application of the tissue engineering products. One of the problems emerging in this area is the relation between resting and migrating cell groups, as well as between different configurations of migrating cells and viscoelasticity. A deeper comprehension of the relation between various configurations of migrating cells and viscoelasticity at the supracellular level represents the prerequisite for optimization of the performance of the artificial epithelium. Since resting and migrating cell groups have a considerable difference in stiffness, a change in their mutual volume ratio and distribution may affect the viscoelasticity of multicellular surfaces. If those cell groups are treated as different phases, then an analogous model may be applied to represent such systems. In this work, a two-step Eyring model is developed in order to demonstrate the main mechanical and biochemical factors that influence configurations of migrating cells. This model could be also used for considering the long-time cell rearrangement under various types of applied stress. The results of this theoretical analysis point out the cause-consequence relationship between the configuration of migrating cells and rheological behavior of multicellular surfaces. Configuration of migrating cells is influenced by mechanical and biochemical perturbations, difficult to measure experimentally, which lead to uncorrelated motility. Uncorrelated motility results in (1) decrease of the volume fraction of migrating cells, (2) change of their configuration, and (3) softening of multicellular surfaces.

## 1. Introduction

One of the key challenges in tissue engineering is to consider tissue remodeling by collective cell migration in response to applied stress and simulate a tissue natural environment under *in vitro* conditions [1–3]. Deeper understanding of long-time cell rearrangement is a prerequisite in the development of functional soft tissue for potential applications in disease modeling and replacing damaged tissues [4]. The intact epithelium plays an important role in the functioning of various organs, and its ability to remodel under various stress conditions would define the level of success in tissue engineering of some organs such as the bladder and the skin.

The main goal of this contribution is to consider cell long-time rearrangement via collective cell migration under stress conditions such as (1) cell aggregate rounding after uniaxial compression between parallel plates [5, 6] and (2) cell aggregate flow subjected to one-dimensional stretching forces using micropipette aspiration [7]. In both cases, cell long-time rearrangement is influenced by external stress, locally or globally. It occurs via collective cell migration within the aggregate 3D surface region or its part driven by tissue surface tension. Consequently, induced volumetric and surface changes could be described by the Young-Laplace law [6]. These systems are analyzed from the standpoint of bionic, as the science that is formed from the combination of various natural and engineering science concepts [8]. Consequently, we discussed the fundamental interrelations between configuration changes of migrating cells and viscoelasticity of multicellular systems at the macroscopic level. Deeper understanding of the multiscale nature of viscoelasticity is necessary in designing the optimal performances of artificial epithelium.

Cell relaxations during and after applying stress occur at various time scales. The time scale of minutes corresponds to single-cell relaxation primarily by adaptation of adhesion complexes while the time scale of hours corresponds to collective cell migration. Guevorkian et al. considered the cell aggregate flow inside the pipette under pressure [7]. They indicated that the cell aggregate responds via short- and long-time pulsated contractions. Short-time contractions correspond to a few minutes and are induced by single-cell contractions. The long-time contractions correspond to tens of minutes and are induced by collective cell migration. These long-time pulsated contractions could be correlated with a change in the configuration of migrating cells. Cell aggregate compression between parallel plates also provokes the organized pattern of cell migration during aggregate rounding in order to minimize the aggregate surface free energy [5, 6, 9–12]. Pajic-Lijakovic and Milivojevic [13] modeled the experimental data of Mombach et al. [5] and pointed that aggregate shape changes take place during successive long-time relaxation cycles. These cycles have various relaxation rates per cycle. The relaxation rates per cycles are not random, but they have a tendency to gather around two or three values indicating an organized cell migration pattern. Every relaxation rate could be related to the various scenarios of cell migration. Three scenarios were considered as follows: (1) most of the cells migrate all the time, (2) some cell groups migrate while the others (at the same time) stay in the resting state, and (3) cells have successive migrating and resting periods in which most of the cells firstly migrate and then stay in the resting state. The average duration of the single relaxation cycle is about 1-2 h [13]. We correlated these scenarios with various configurations of migrating cells. Mombach et al. pointed to exponential changes in the aggregate shape from ellipsoidal to spherical [5]. Consequently, the linear nature of long-time cell rearrangement obtained experimentally at a macroscopic level has been modeled by applying the Eyring transition state theory by Marmottant et al. [6] and Pajic-Lijakovic and Milivojevic [13]. Cell surface rearrangement could be treated as a thermodynamic system close to equilibrium at the macroscopic level. However, cell surface rearrangement considered at a mesoscopic level has been treated as thermodynamic systems far from equilibrium [14]. It is in accordance with the fact that internal fluctuations, which are significant during thermodynamic system structural ordering at the mesoscopic level, could be neglected at the macroscopic level [15].

Viscoelasticity depends on the configurations of migrating cells. Migrating cell clusters are much stiffer than resting ones due to the accumulation of contractile energy. These contractions induce the generation of prestress. Lange and Fabry reported that cytoskeletal prestress causes adherent cells to stiffen [16]. Lange and Fabry reported that muscle cells can change their elastic modulus by over one order of magnitude from less than 10 kPa in a relaxed (resting) state to around 200 kPa in a fully activated (migrating) state [16]. Consequently, the multicellular surfaces could be treated as a two-phase pseudoblend from the mechanical standpoint [14]. The migrating pseudophase represents the dispersion within the resting one. The influence of configurations of migrating cells on the viscoelasticity of multicellular systems at a mesoscopic level has been discussed in the context of the mechanical coupling modes [14]. They reported that cell migration within a large number of small clusters corresponds to series mode coupling, while cell migration as monolayer sheets corresponds to parallel mode coupling. Consequently, mode coupling should be related to the biointerface size between migrating and resting cell pseudophases.

The shape of migrating cell groups could vary from small cell clusters to monolayer sheets depending on cell types and microenvironmental conditions [17–19]. Mikami et al. discussed collective cell migration of stratified epithelial cells toward the wound in the form of monolayer sheets [20]. All epithelial cells within the sheet migrate, maintaining cell-cell adhesions [21]. Migrating cell sheets slide over the surrounding cell layers in the resting state [18, 22]. The number of sheets and their sizes depend on the size, shape, and depth of injury [22]. Cell organization in the form of migrating cell sheets and their sliding over the surrounding unperturbed cell layers of epithelium pointed to the ordered lamellar structure. Friedl and Alexander considered collective cell migration during cancer invasion and metastases [17]. They concluded that some tumor types could migrate within partially connected strands through surrounding tissue while others could migrate in the forms of monolayer sheets or small cell clusters. Some other cell types could also migrate within small clusters through surrounding tissue [17].

We expand previous considerations proposed by Pajic-Lijakovic and Milivojevic [13] and formulate modified a two-step Eyring model for describing (1) resting-to-migrating cell state transition and vice versa and (2) configuration changes of migrating cells from small clusters to monolayer sheets. Obtained configuration changes of migrating cells should be related to the viscoelasticity of the multicellular surface based on mechanical coupling modes. Pajic-Lijakovic and Milivojevic [14] considered cell surface rearrangement at a mesoscopic level and proposed (1) series mode coupling for the surface parts in which cell migrates in the form of small clusters and (2) parallel mode coupling for the surface parts in which cell migrates in the form of monolayer sheets. Here, we expand this consideration obtained at the mesoscopic level to the macroscopic level by formulating mixed, series-parallel, mode coupling. Mixed mode coupling accounts for both fractions of cells (migrating and resting) coupled in series and in parallel.

## 2. Phenomenological Background of the Model Based on Experiments of Cell Aggregate Rounding

Experimental data for the aggregate shape relaxation after uniaxial compression, considered here, shows the important feature obtained from the data fluctuations. These fluctuations clearly point to an ordered relaxation trend in the form of successive relaxation cycles. The ordered fluctuation trend in the form of long-time pulsated contractions was also obtained during cell aggregate flow inside the pipette under pressure [7]. Accordingly, the aggregate shape long-time relaxation after compression for the *j*^th^ cycle has been expressed by Pajic-Lijakovic and Milivojevic as *ε*(*t*)^*j*^ = *ε*^*j*^_0_*e*^−*k*^*j*^*t*^ (where *ε*(*t*)^*j*^ is the deformation parameter for *t* ∈ [0, Δ*t*^*j*^] during the *j*^th^ relaxation cycle equal to *ε*(*t*) = AR(*t*) − 1, AR(*t*) is the aggregate aspect ratio, *ε*^*j*^*o* is the initial value for the deformation parameter, and *k*^*j*^ is the relaxation rate for the *j*^th^ cycle) [13]. The relaxation rates are not random but grouped around two or three values indicating an organized cell migrating pattern: (1) *k*_m_, most of the cells migrate (the volume fraction of migrating cells is *ϕ*_m_ → 1), (2) *k*_r_ ≈ 0, most of the cells stay in the resting state (the volume fraction of resting cells is *ϕ*_r_ → 1), and (3) *k*_t_, some cell groups migrate while the others, at the same time, stay in the resting state. The relaxation rate per cycle should be related to the volume fraction and configuration of migrating cells, i.e., *k*^*j*^ = *k*^*j*^ (*ϕ*_m_^*j*^, configuration^*j*^). However, the formulation of this relationship needs the additional surface characterization as the surface stiffness distribution and the rate of its change.

A significant difference in cell stiffness between migrating and resting cell groups indicates that volume fraction of migrating cells and their distribution could influence the long-time rheological behavior of multicellular surfaces. This aspect of cell surface rearrangement could be treated by the analogy with physics in the form of a two-phase blend composed of migrating and resting cell pseudophases. Migrating pseudophase could form various configurations: (1) small clusters, (2) monolayer sheets, and (3) mixed configurations composed of both dispersion of small clusters and lamellar structural parts [17, 21]. For mixed configurations, the volume fraction of migrating cells *ϕ*_m_(*t*) could be expressed as follows:

(1) $$\begin{array}{c} \phi_{\text{m}}\left(t\right)=\phi_{\text{Sm}}\left(t\right)+\phi_{\text{Pm}}\left(t\right), \end{array}$$

where *ϕ*_Sm_(*t*) is the part of migrating cells in the form of small clusters equal to *ϕ*_Sm_(*t*) = *N*_Sm_(*t*)/*N* and *ϕ*_Pm_(*t*) is the part of migrating cells in the form of monolayer sheets equal to *ϕ*_Pm_(*t*) = *N*_Pm_(*t*)/*N* and *N* is the number of cells in the surface region. Cell aggregate compression induces the perturbation of the aggregate surface region consisting of *N* active cells which undergo to short- and long-time relaxations. Short-time relaxation describes relaxation of cell volumes and cell packing state within the surface region (the time scale of minutes) [5]. Long-time relaxation describes surface relaxation caused by collective cell migration (the time scale of hours). Surface tension is the main mechanism which influences the aggregate rounding. It represents the “driving force” for collective cell migration. Some of the active cells within this region become active and migrate in order to decrease the aggregate surface as well as the surface free energy. Cell packing density and cell volumes relax quickly and become constant during aggregate rounding. Long-time relaxation leads to change of the thickness and surface of the surface region while the volume remains constant. Total number of cells in the surface region consists of migrating and resting cells. The average volume fraction of resting cells is equal to

(2) $$\begin{array}{c} \phi_{\text{r}}\left(t\right)=1-\phi_{\text{m}}\left(t\right), \end{array}$$

where the volume fraction of resting cells is equal to *ϕ*_Pr_(*t*) + *ϕ*_Sr_(*t*) = *ϕ*_r_(*t*), while *ϕ*_Sr_(*t*) = *N*_Sr_(*t*)/*N* is the volume fraction of resting cells in the surroundings of migrating cells *ϕ*_Sm_(*t*) and *ϕ*_Pr_(*t*) = *N*_Pr_(*t*)/*N* is the volume fraction of resting cells in the surroundings of migrating cells *ϕ*_Pm_(*t*).

The resting pseudophase is in the surroundings of the migrating pseudophase for both configurations. Higher volume fraction *ϕ*_Pm_(*t*) corresponds to (1) higher volume fraction *ϕ*_Pr_(*t*) and (2) lower volume fractions *ϕ*_Sm_(*t*) and *ϕ*_Sr_(*t*). When the volume fraction is (1) *ϕ*_Pm_(*t*) → 0, it means that *ϕ*_Pr_(*t*) → 0, and/or when the volume fraction is (2) *ϕ*_Sm_(*t*) → 0, it means that *ϕ*_Sr_(*t*) → 0. Consequently, we can correlate the volume fraction of the resting pseudophase with the volume fraction of the migrating pseudophase for both configurations in the form of additional condition as

(3) $$\begin{array}{c} \frac{\phi_{\text{Pr}}\left(t\right)}{\phi_{\text{Sr}}\left(t\right)}=\frac{\phi_{\text{Pm}}\left(t\right)}{\phi_{\text{Sm}}\left(t\right)}, \end{array}$$

which offers the possibility of formulating the volume fractions of resting cells for both configurations as *ϕ*_Sr_(*t*) = *ϕ*_r_(*t*)(1/1 + *X*(*t*)) and *ϕ*_Pr_(*t*) = *ϕ*_r_(*t*)(*X*(*t*)/1 + *X*(*t*)), while the model parameter *X*(*t*) is equal to *X*(*t*) = *ϕ*_Pm_(*t*)/*ϕ*_Sm_(*t*).

Configuration changes of a migrating cell pseudophase take place in order to minimize the interface size between the resting and migrating cell pseudophases. Higher interface size leads to intensive mechanical and biochemical perturbations caused by uncorrelated motility [23]. In the initial phase of cell rearrangement during aggregate rounding, migrating cells form small clusters (*ϕ*_m_(*t*) = *ϕ*_m_^*L*^(*t*)≺≺1, *ϕ*_m_^*L*^(*t*) ≈ *ϕ*_Sm_(*t*) while *ϕ*_Pm_(*t*) → 0). However, the increase of the volume fraction of migrating cells leads to increase of the interface size. In order to reduce the interface size as well as the surface free energy, migrating cells could form monolayer sheets instead of small clusters. Consequently, for high volume fraction of migrating cell (*ϕ*_m_(*t*) = *ϕ*_m_^*H*^(*t*) → 1), cell migration in the form of monolayer sheets becomes dominant, i.e., *ϕ*_m_^*H*^(*t*) ≈ *ϕ*_Pm_(*t*).

**Figure 1:**
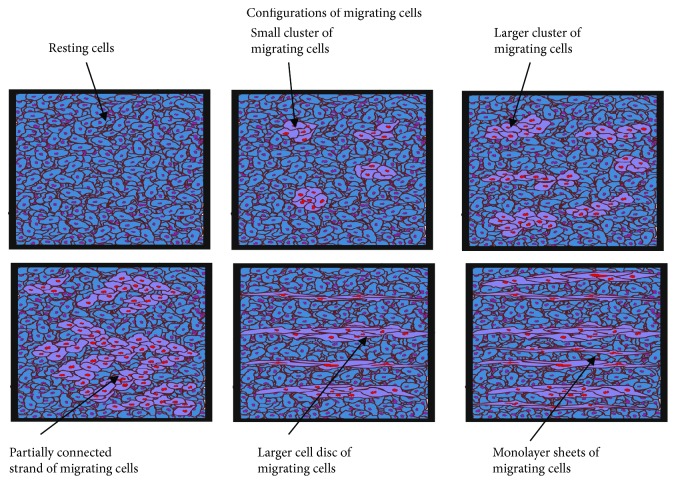
Schematic representation of various configurations of migrating cells.

Cell rearrangement could be described by three variables: (1) volume fraction of resting cells *ϕ*_r_(*t*), (2) volume fraction of migrating cells in the form of small clusters *ϕ*_Sm_(*t*), and (3) volume fraction of migrating cells in the form of monolayer sheets *ϕ*_Pm_(*t*) and could be treated as a two-step process. Configuration changes of migrating cells were shown schematically in Figure 1.

The increase of the volume fraction of migrating cells leads to configuration changes from migration within small cell clusters to migration within monolayer sheets. It is in accordance with minimizing the biointerface between the migrating and resting cell pseudophases as well as the surface free energy.

## 3. The Two-Step Model Development

Configuration changes of migrating cells could be treated as two-step processes. Both steps are reversible and could be described by applying transition state theory in the form of Eyring modeling approach which has been already applied for describing cell rearrangement [6, 13]. The first step represents the resting-to-migrating cell state transition and vice versa, while the second one represents the configuration changes of migrating cells from dispersed small clusters to monolayer sheets and vice versa. The two-step model could be expressed as

(4) $$\begin{array}{c} R\overset{k_{2}}{\underset{k_{1}}{⇆}}\text{Ms}\overset{k_{4}}{\underset{k_{3}}{⇆}}\text{Mp}, \end{array}$$

where *R* represents the resting pseudophase, Ms is the migrating cells in the form of small clusters coupled in series, Mp is the migrating cells coupled in parallel, *k*_1_ is the rate of *R* → Ms cell state transition, *k*_2_ is the rate of Ms → *R* cell state transition, *k*_3_ is the rate of Ms → Mp cell state transition, and *k*_4_ is the rate of Mp → Ms cell state transition. Kinetic constants should be related to the mechanisms of phase transitions. Detailed description of the kinetic constants will be described in the next section.

The two-step model treated cells within the surface regions as a canonical ensemble. Modeling equations describe transitions (1) from *N*_r_(*t*) to *N*_Sm_(*t*) and vice versa and (2) from *N*_Sm_(*t*) to *N*_Pm_(*t*) and vice versa, while *N* = const. as shown in Appendix A. For this condition, modeling equations which correlate the volume fractions of pseudophases for both configurations could be formulated as

(5) $$\begin{array}{c} \frac{d\phi_{\text{r}}\left(t\right)}{dt}=-k_{1}\phi_{\text{r}}\left(t\right)+k_{2}\phi_{\text{Sm}}\left(t\right),\\ \frac{d\phi_{\text{Sm}}\left(t\right)}{dt}=k_{1}\phi_{\text{r}}\left(t\right)-\left(k_{2}+k_{3}\right)\phi_{\text{Sm}}\left(t\right)+k_{4}\phi_{\text{Pm}}\left(t\right),\\ \frac{d\phi_{\text{Pm}}\left(t\right)}{dt}=k_{3}\phi_{\text{Sm}}\left(t\right)-k_{4}\phi_{\text{Pm}}\left(t\right), \end{array}$$

with an initial condition, at *t* = 0, all cells within the surface are in the resting state, i.e., the volume fractions are equal to *ϕ*_r_(0) = 1 and *ϕ*_Sm_(0) = *ϕ*_Pm_ = 0. The solution of model equations is expressed as [24]

(6) $$\begin{array}{c} \phi_{\text{r}}\left(t\right)=\phi_{\text{r}}\left(0\right)\left[1-k_{1}\left(\frac{k_{1}+k_{3}}{\lambda_{1}\lambda_{2}}-\frac{k_{3}-\lambda_{1}+k_{4}}{\lambda_{1}\left(\lambda_{2}-\lambda_{1}\right)}e^{-\lambda_{1}t}+\frac{k_{4}-\lambda_{2}+k_{3}}{\lambda_{2}\left(\lambda_{2}-\lambda_{1}\right)}e^{-\lambda_{2}t}\right)\right],\\ \phi_{\text{Sm}}\left(t\right)=\phi_{\text{r}}\left(0\right)k_{1}\left[\frac{k_{4}}{\lambda_{1}\lambda_{2}}-\frac{k_{4}-\lambda_{1}}{\lambda_{1}\left(\lambda_{2}-\lambda_{1}\right)}e^{-\lambda_{1}t}+\frac{k_{4}-\lambda_{2}}{\lambda_{2}\left(\lambda_{2}-\lambda_{1}\right)}e^{-\lambda_{2}t}\right],\\ \phi_{\text{Pm}}\left(t\right)=\phi_{\text{r}}\left(0\right)k_{1}k_{3}\left[\frac{1}{\lambda_{1}\lambda_{2}}-\frac{1}{\lambda_{1}\left(\lambda_{2}-\lambda_{1}\right)}e^{-\lambda_{1}t}+\frac{1}{\lambda_{2}\left(\lambda_{2}-\lambda_{1}\right)}e^{-\lambda_{2}t}\right], \end{array}$$

where the model parameters *λ*_1_ and *λ*_2_ could be determined from the relations *λ*_1_*λ*_2_ = *k*_2_*k*_4_ + *k*_1_*k*_3_ + *k*_1_*k*_4_ and *λ*_1_ + *λ*_2_ = *k*_1_ + *k*_2_ + *k*_3_ + *k*_4_. Model parameters *λ*_1_ and *λ*_2_ have real solutions for the condition (*k*_2_*k*_4_ + *k*_1_*k*_3_ + *k*_1_*k*_4_)^2^ − 4(*k*_1_ + *k*_2_ + *k*_3_ + *k*_4_) ≥ 0. The volume fraction of resting cells consists of two subpopulations: (1) resting cells in the surroundings of small migrating cell clusters and (2) resting cells in the surroundings of migrating monolayer sheets. The state of the multicellular surface depends on the values of kinetic constants *k*_1_, *k*_2_, *k*_3_, *k*_4_.

The choice of kinetic constants defines the various scenarios of long-time cell surface rearrangement and the corresponding equilibrium configurations of migrating cells described by volume fractions *ϕ*_Sm_^eq^, *ϕ*_Pm_^eq^, *ϕ*_Sr_^eq^, *ϕ*_Pr_^eq^ Equilibrium volume fractions are determined for the condition *t* → ∞ and expressed as:

(7) $$\begin{array}{c} {\phi_{\text{Sm}}}^{eq}=k_{1}\frac{k_{4}}{\lambda_{1}\lambda_{2}},\\ {\phi_{\text{Pm}}}^{\text{eq}}=k_{1}k_{3}\frac{1}{\lambda_{1}\lambda_{2}},\\ {\phi_{\text{Sr}}}^{\text{eq}}=\left(1-k_{1}\frac{k_{1}+k_{3}}{\lambda_{1}\lambda_{2}}\right)\frac{1}{1+X_{\text{eq}}},\\ {\phi_{\text{Pr}}}^{\text{eq}}=\left(1-k_{1}\frac{k_{1}+k_{3}}{\lambda_{1}\lambda_{2}}\right)\frac{X_{\text{eq}}}{1+X_{\text{eq}}}, \end{array}$$

where *X*_eq_ = *k*_3_. This model is formulated for 3D systems. However, it could be also applied to 2D systems. In this case, surface fractions of cells should be used instead of volume fraction of cells in the modeling equations.

### 3.1. Kinetic Constant Formulation Depending on the Uncorrelated Motility

Cell rearrangement, described by the proposed model equation (5), could be managed by the kinetic constants *k*_1_, *k*_2_, *k*_3_, *k*_4_. Resting-to-migrating cell state transition is induced by local stress accumulation, while changes in migrating cell configuration (from dispersed small clusters to monolayer sheets) are induced by the cohesiveness inhomogeneity. External stress ${\tilde{\sigma }}_{\mathrm{ext}}$ provokes internal stress accumulation $\Delta {\tilde{\Pi }}_{\text{eff}}$ (effective “mechanical driving force”) which leads to resting-to-migrating cell state transition. It represents the first step of this complex process. In the second step, inhomogeneity of cell cohesiveness Δ*E*_int_ provokes cell biomechanical response related to cell signaling Δ*E*_*eff*_ (effective “biochemical driving force”) which leads to configuration changes of migrating cells from small clusters to monolayer sheets. Kinetic constants should be formulated in the context of the transition state theory by formulating corresponding energy barriers. The mechanical barrier is formulated in Appendix B while the biochemical barrier is formulated in Appendix C.

The rate constants *k*_1_ and *k*_2_ are expressed as

(8) $$\begin{array}{c} k_{1}=k^{I}e^{-\left({\tilde{\sigma }}_{\mathrm{ext}}-\Delta {\tilde{\Pi }}_{\text{eff}}\right)\cdot f^{-1}\left(\lambda_{E}\tilde{Z}\right)},\\ k_{2}=k^{I}e^{-\left({\tilde{\sigma }}_{\mathrm{ext}}+\Delta {\tilde{\Pi }}_{\text{eff}}\right)\cdot f^{-1}\left(\lambda_{E}\tilde{Z}\right)}, \end{array}$$

where *k*^*I*^ is the frequency, $f^{-1}\left(\lambda_{E}\tilde{Z}\right)$ is the inverse map, the effective mechanical driving force is equal to Δ*Π*_eff *ij*_ = Δ*Π*_r−m *ij*_ − Δ*Π*_P *ij*_, Δ*Π*_r−m *ij*_ is the stress difference between migrating and resting cell pseudophases at the biointerface, and Δ*Π*_P *ij*_ is the perturbation stress component which is approximately equal to the unit stress, i.e., Δ*Π*_P *ij*_ ≈ *λ*_*E*_*Z*_*ij*_. The number of stress quanta for resting-to-migrating cell state transition is equal to${n_{\text{stress}}}^{+}=\left({\tilde{\sigma }}_{\mathrm{ext}}-\Delta {\tilde{\Pi }}_{\text{eff}}\right)\cdot f^{-1}\left(\lambda_{E}\tilde{Z}\right)$, while the number of quanta for migrating-to-resting cell state transition is equal to ${n_{\text{stress}}}^{-}=\left({\tilde{\sigma }}_{\mathrm{ext}}+\Delta {\tilde{\Pi }}_{\text{eff}}\right)\cdot f^{-1}\left(\lambda_{E}\tilde{Z}\right)$. The rate constant *k*_1_ for resting-to-migrating cell state transition is supposed to be equal to *k*_1_ ~ *t*_p_^−1^ (where *t*_p_ is the cell persistence time). McCann et al. reported that cell persistence time corresponds to several tens of minutes [25].

The ratio *k*_1_/*k*_2_ is equal to *k*_1_/*k*_2_ = *e*^Δ*n*_stress_^ (where Δ*n*_stress_ = *n*_stress_^−^ − *n*_stress_^+^ and Δ*n*_stress_ ≥ 0). Three cases of the ratio *k*_1_/*k*_2_ could be considered depending on Δ*n*_stress_:

1. Δ*n*_stress_≻0 (and Δ*Π*_r−m *ij*_≻Δ*Π*_p *ij*_) which corresponds to *k*_1_≻*k*_2_
2. Δ*n*_stress_ ≈ 0 (and Δ*Π*_r−m *ij*_ ≈ Δ*Π*_p *ij*_) which corresponds to *k*_1_ ~ *k*_2_
3. Δ*n*_stress_≺0 (and Δ*Π*_r−m *ij*_≺Δ*Π*_p *ij*_) which corresponds to *k*_1_≺*k*_2_

The rates *k*_3_ and *k*_4_ are expressed as:

(9) $$\begin{array}{c} k_{3}=k^{II}e^{-\left(\left(\Delta E_{\mathrm{int}}-\Delta E_{\text{eff}}\right)/k_{B}T_{\text{eff}}\right)},\\ k_{4}=k^{II}e^{-\left(\left(\Delta E_{\mathrm{int}}+\Delta E_{\text{eff}}\right)/k_{B}T_{\text{eff}}\right)}, \end{array}$$

where *k*^*II*^ is the frequency, Δ*E*_int_ is the biochemical barrier equal to Δ*E*_int_ = *γ*_r_Δ*A*_m_ − (*γ*_m_ + *γ*_int_)Δ*A*_m_, *γ*_r_ is the surface tension of the resting cell pseudophase, *γ*_m_ is the surface tension of the migrating cell pseudophase, *γ*_int_ is the interfacial tension, Δ*A*_m_ is the surface change of the migrating cell groups, and Δ*E*_eff_ is the effective “biochemical driving force, i.e., the cohesiveness difference between the pseudophases” which leads to configuration changes of migrating cells from small clusters to monolayer sheets, *k*_*B*_ is the Boltzmann constant, and *T*_eff_ is the effective temperature. Kinetic constants *k*_3_ and *k*_4_ account for the cohesiveness difference. As was shown, the stress barrier for the first-step process was defined relative to the unit of stress. Similarly, the energy barrier for the second-step process should be defined relative to the unit of energy. This unit of energy should be related to cellular mobility as the main cellular characteristic responsible to long-time rearrangement. By the analogy with the thermal unit of energy expressed as *∆E*_*T*_ = *k*_*B*_*T*, we define the cellular long-time rearrangement energy unit as *∆E*_*C*_ = *k*_*B*_*T*_eff_ (where *T*_eff_ is the effective temperature). The concept of effective temperature has been applied for considering rearrangement of various thermodynamic systems (close to equilibrium and far from equilibrium) from glasses and sheared fluids to granular systems [26]. We applied this concept to the long-time rearrangement of dense cellular systems. The number of interfacial energy quanta for configuration changes of migrating cells from small clusters to monolayer sheets is equal to *n*_int_^+^ = Δ*E*_int_ − Δ*E*_eff_/*k*_*B*_*T*_eff_, while the number of interfacial energy quanta for configuration changes of migrating cells from monolayer sheets to small clusters is equal to *n*_int_^−^ = Δ*E*_int_ + Δ*E*_eff_/*k*_*B*_*T*_eff_.

The ratio *k*_3_/*k*_4_ is equal to *k*_3_/*k*_4_ = *e*^Δ*n*_int_^ (where Δ*n*_int_ = *n*_int_^−^ − *n*_int_^+^ and Δ*n*_int_ ≥ 0). Three causes of the ratio *k*_3_/*k*_4_ could be considered depending on Δ*n*_int_:

1. Δ*n*_int_≻0 (and Δ*E*_S_≻Δ*E*_P_) which corresponds to *k*_3_≻*k*_4_
2. Δ*n*_int_ ≈ 0 (and Δ*E*_S_ ≈ Δ*E*_P_) which corresponds to *k*_3_ ~ *k*_4_
3. Δ*n*_int_≺0 (and Δ*E*_S_≺Δ*E*_P_) which corresponds to *k*_3_≺*k*_4_

The ratio *k*_1_/*k*_3_ is *k*_1_/*k*_3_ = *e*^*n*_int_^+^−*n*_stress_^+^^. Three causes of the ratio *k*_1_/*k*_3_ could be considered:

1. If *n*_int_^+^≻*n*_stress_^+^ indicates that *k*_1_≻*k*_3_
2. If *n*_int_^+^ ≈ *n*_stress_^+^ indicates that *k*_1_ ≈ *k*_3_
3. If *n*_int_^+^≺*n*_stress_^+^ indicates that *k*_1_≺*k*_3_

The ratio *k*_2_/*k*_4_ is *k*_2_/*k*_4_ = *e*^*n*_int_^−^−*n*_stress_^−^^. Three causes of the ratio *k*_2_/*k*_4_ could be considered:

1. If *n*_int_^−^≻*n*_stress_^−^ indicates that *k*_2_≻*k*_4_
2. If *n*_int_^−^ ≈ *n*_stress_^−^ indicates that *k*_2_ ≈ *k*_4_
3. If *n*_int_^−^≺*n*_stress_^−^ indicates that *k*_2_≺*k*_4_

Consequently, two conditions are necessary for defining the single state of the multicellular surface. Two constants are related per single condition. We formulated 12 conditions placed within 3 groups. Combinations within groups could not define a new system state. The number of states could be expressed as *n*_state_ = *C*_2_^12^ − *C*_2_^3^ = 54 (where *n*_state_ is the number of states, *C*_2_^12^ is the combination of 12 conditions of the second class, and *C*_2_^3^ is the combination of 3 conditions of the second class).

These 3 groups of cases determine the influence of uncorrelated motility on cell resting-to-migrating cell state transition and vice versa as well as configuration changes of migrating cells through mechanical perturbations Δ*Π*_*P* *ij*_ and biochemical perturbations Δ*E*_*P*_. Two cases (first group case 1 and second group case 1) correspond to low mechanical and biochemical perturbations, respectively. Two cases (first group case 2 and second group case 2) correspond to medium mechanical and biochemical perturbations. Two cases (first group case 3 and second group case 3) correspond to large mechanical and biochemical perturbations. Uncorrelated motility accounts for internal and external effects. Internal effects are caused by stress accumulation within migrating cell groups during their intercalation through a dense cellular environment [23]. External effects represent the consequence of the collision of velocity fronts which is significant even in 2D [27]. Uncorrelated motility represents the main cause of change of the relaxation rate of the aggregate shape after uniaxial compression from cycle to cycle [13]. Additional experiments are needed to correlate uncorrelated motility with cell type and microenvironmental conditions. This modeling consideration accounts for uncorrelated motility and its impact to dynamics of configuration changes.

### 3.2. Viscoelasticity of Multicellular Surface-Mechanical Coupling Modes

The estimation of the volume fractions of pseudophases for both configurations of migrating cells is the prerequisite for the detailed characterization of the viscoelasticity of multicellular surfaces. The viscoelasticity depends on (1) established configurations and volume fraction of migrating cells which influences the biointerface size, (2) stress-strain relations for the migrating cell pseudophase for both configurations, and (3) stress-strain relations for the resting cell pseudophase for both configurations. Biointerface size influences the viscoelasticity through mechanical coupling modes [14].

Pajic-Lijakovic and Milivojevic proposed two types of mode coupling, series and parallel, suitable for consideration on the mesoscopic level [14]. At the mesoscopic level, the biointerface size could be expressed as *dA*_m_(*r*, *t*)/*dV*_m_(*r*, *t*) = 1/*ξ*_*i*_(*r*, *t*) (where *ξ*_*i*_(*r*, *t*) is the local interface dimension, *dA*_m_(*r*, *t*) is the local surface of migrating cell groups, and *dV*_m_(*r*, *t*) is the local volume of migrating cell groups). The low-interface area corresponds to cell migration within a few monolayer sheets. The high interface area, for the same volume fraction of migrating cells, corresponds to cell migration within a large number of small migrating cell groups (in the form of clusters) dispersed within the resting cell pseudophase. The high interface area corresponds to series mode coupling while the small interface area corresponds to parallel mode coupling. For considering surface rearrangement at the macroscopic level, it is necessary to combine both contributions, i.e., series and parallel. We propose mixed, series-parallel mode coupling as suitable for the modeling of macroscopic dynamics. The mixed mode accounts for both series and parallel contributions to some extent as

(10) $$\begin{array}{c} \phi_{\text{P}}\left(t\right)=\phi_{\text{Pm}}\left(t\right)+\phi_{\text{Pr}}\left(t\right),\\ \phi_{\text{S}}\left(t\right)=\phi_{\text{Sm}}\left(t\right)+\phi_{\text{Sr}}\left(t\right), \end{array}$$

where *ϕ*_P_(*t*) is the average volume fraction of the surface part coupling in parallel and *ϕ*_Pm_(*t*) is the average volume fraction of migrating cells in the form of the monolayer, while *ϕ*_Pr_(*t*) is the average volume fraction of the surrounding resting phase. When the migrating cells form the monolayer sheets, this lamellar structure contributes to the viscoelasticity by parallel mode coupling. The average volume fraction of the surface part coupling in series *ϕ*_S_(*t*) is equal to *ϕ*_S_(*t*) = 1 − *ϕ*_P_(*t*). When the migrating cells form a large number of small clusters, this structure contributes to the viscoelasticity by parallel mode coupling. Consequently, *ϕ*_Sm_(*t*) is the average volume fraction of migrating cells in the form of small clusters and *ϕ*_Sr_(*t*) is the average volume fraction of the surrounding resting cells. The average volume fraction of the migrating phase is equal to

(11) $$\begin{array}{c} \phi_{\text{m}}\left(t\right)=\phi_{\text{Sm}}\left(t\right)+\phi_{\text{Pm}}\left(t\right), \end{array}$$

while the average volume fraction of resting phase is equal to *ϕ*_r_(*t*) = 1 − *ϕ*_m_(*t*).

The constitutive rheological model of multicellular systems for cell long-time rearrangement depends on the cell type and the intensity and the way of applying stress. Consequently, multicellular systems can behave as viscoelastic liquid or viscoelastic solid. Guevorkian et al. proposed the Maxwell model for describing the cellular flow inside the pipette under pressure at the time scale of several tens of minutes [7]. They applied pressure locally in the range of 0.5 kPa to 1.2 kPa. These experimental conditions induced intensive energy dissipation during cell long-time rearrangement. The Maxwell model suitable for viscoelastic liquid points that stress relaxes under constant strain rate conditions, while strain itself cannot relax. Consequently, this model represents a good choice as long as the equilibrium strain which corresponds to the equilibrium pipette length filled with cells is not observed. Joanny and Prost also proposed the Maxwell model for describing the long-time cell rearrangement [28]. They elaborated their assumption on the basis of the work reported by Wottawah et al. [29]. However, Wottawah et al. considered single-cell stretching during a few minutes and proposed the linear three-parameter model rather than the Maxwell model [29]. Chen et al. pointed to the nematic property of the cell monolayer during cell long-time rearrangement [30]. Iordan et al. [31] and Preziosi et al. [32] considered short-time viscoelasticity of cell suspensions in the wide range of cell volume fraction within two types of experimental conditions: (1) under low oscillator strain within a frequency range from 10^−1^ to 10 Hz and (2) under a wide range of shear rates from 10^−3^ to 10^3^ s^−1^. This frequency range corresponds to characteristic times from 0.1 to 10 s. Preziosi et al. proposed the Maxwell model for short-time viscoelasticity [32]. Iordan et al. reported that short-time rheological response under oscillator strain conditions corresponds to viscoelastic solid for volume fraction of cells higher than 20% [31]. It is in accordance with the fact that storage modulus is higher than loss modulus. However, in this paper, we consider long-time viscoelasticity caused by collective cell migration obtained at the time scale of several tens of minutes to hours based on the proposed model.

Cell aggregate rounding after uniaxial compression corresponds to cell long-time rearrangement caused by collective cell migration. The results point that the (1) cell aggregate shape relaxes, (2) aggregate surface relaxes, and (3) aggregate surface strain also relaxes. The ability of surface strain to relax pointed to viscoelastic solid rather than viscoelastic liquid. On the contrary, Flenner et al. treated cell aggregate rounding as viscoelastic liquid [33]. They introduced two interconnected arguments: (1) cell aggregate rounding is driven by surface tension and (2) the surface tension represents the characteristic of liquid. We agree that aggregate rounding is driven by tissue surface tension. However, surface tension is not necessarily the characteristic of liquid. Amorphous viscoelastic solids such as polymer hydrogels and foams also have surface tension [34].

For the mixed coupling mode, the multicellular surface stress and strain are formulated by the modified model proposed by Takayanagi et al. [35] and modified by Kolarik et al. [36] for the multicomponent polymer blend. The model for the mixed coupling mode is expressed as

(12) $$\begin{array}{c} {\tilde{\sigma }}_{\text{surface}}\left(t\right)=\phi_{\text{P}}\left(t\right){\tilde{\sigma }}_{\text{P}}\left(t\right)+\left(1-\phi_{\text{P}}\left(t\right)\right){\tilde{\sigma }}_{\text{S}}\left(t\right),\\ {\tilde{\varepsilon }}_{\text{surface}}\left(t\right)={\tilde{\varepsilon }}_{\text{P}}\left(t\right)={\tilde{\varepsilon }}_{\text{S}}\left(t\right), \end{array}$$

where ${\tilde{\sigma }}_{\text{surface}}\left(t\right)$ is the surface stress tensor and ${\tilde{\sigma }}_{\text{P}}\left(t\right)$ is the stress part for the corresponding parallel coupling. ${\tilde{\sigma }}_{\text{S}}\left(t\right)$ is the stress part for the corresponding series coupling, ${\tilde{\varepsilon }}_{\text{surface}}\left(t\right)$ is the surface strain tensor, ${\tilde{\varepsilon }}_{\text{P}}\left(t\right)$ is the strain part for the corresponding parallel coupling, ${\tilde{\varepsilon }}_{\text{S}}\left(t\right)$ is the strain coupling, and ${\tilde{\varepsilon }}_{\text{P}}\left(t\right)$ is the strain part for the corresponding series coupling. The stress tensor ${\tilde{\sigma }}_{\text{P}}\left(t\right)$ is equal to

(13) $$\begin{array}{c} \phi_{\text{P}}\left(t\right){\tilde{\sigma }}_{\text{P}}\left(t\right)=\phi_{\text{Pm}}\left(t\right){\tilde{\sigma }}_{\text{Pm}}\left(t\right)+\phi_{\text{Pr}}\left(t\right){\tilde{\sigma }}_{\text{Pr}}\left(t\right), \end{array}$$

where ${\tilde{\sigma }}_{\text{Pm}}\left(t\right)$ and ${\tilde{\sigma }}_{\text{Pr}}\left(t\right)$ are the stresses for the migrating phase and resting phase parts coupled in parallel. The strain tensor ${\tilde{\varepsilon }}_{\text{P}}\left(t\right)$ is equal to

(14) $$\begin{array}{c} {\tilde{\varepsilon }}_{\text{P}}\left(t\right)={\tilde{\varepsilon }}_{\text{Pm}}\left(t\right)={\tilde{\varepsilon }}_{\text{Pr}}\left(t\right), \end{array}$$

where ${\tilde{\varepsilon }}_{\text{Pm}}\left(t\right)$ and ${\tilde{\varepsilon }}_{\text{Pr}}\left(t\right)$ are the local strains for the migrating phase and resting phase parts coupled in parallel. The stress tensor ${\tilde{\sigma }}_{\text{S}}\left(t\right)$ is equal to

(15) $$\begin{array}{c} {\tilde{\sigma }}_{\text{S}}\left(t\right)={\tilde{\sigma }}_{\text{Sm}}\left(t\right)={\tilde{\sigma }}_{\text{Sr}}\left(t\right), \end{array}$$

where ${\tilde{\sigma }}_{\text{Sm}}\left(t\right)$ and ${\tilde{\sigma }}_{\text{Sr}}\left(t\right)$ are the stresses for the migrating phase and resting phase parts coupled in series. The strain tensor ${\tilde{\varepsilon }}_{\text{S}}\left(t\right)$ is equal to

(16) $$\begin{array}{c} \phi_{\text{S}}\left(t\right){\tilde{\varepsilon }}_{\text{S}}\left(t\right)=\phi_{\text{Sm}}\left(t\right){\tilde{\varepsilon }}_{\text{Sm}}\left(t\right)+\phi_{\text{Sr}}\left(t\right){\tilde{\varepsilon }}_{\text{Sr}}\left(t\right), \end{array}$$

where ${\tilde{\varepsilon }}_{\text{Sm}}\left(t\right)$ and ${\tilde{\varepsilon }}_{\text{Sr}}\left(t\right)$ are the local strains for the migrating phase and resting phase parts coupled in series.

Detailed consideration of time-dependent rheological response of multicellular systems required additional knowledge of constitutive models for migrating cell groups and surrounding resting cells. We will present the simplified consideration of current equilibrium states based on the equilibrium configurations of migrating cells, expressed by volume fractions *ϕ*_Sm_^eq^, *ϕ*_Pm_^eq^, *ϕ*_Sr_^eq^, *ϕ*_Pr_^eq^. Consequently, the modeling results could be characterized from the stand point of the surface stiffness in the context of the mixed coupling mode. The surface stiffness could be quantified by the complex modulus of the cell surface $G^{*}\left(\omega \right)=F\left({\tilde{\sigma }}_{\text{surface}}\left(t\right)\right)/F\left({\tilde{\varepsilon }}_{\text{surface}}\left(t\right)\right)$, the complex modulus of the migrating pseudophase ${G_{m}}^{*}\left(\omega \right)=F\left({\tilde{\sigma }}_{m}\left(t\right)\right)/F\left({\tilde{\varepsilon }}_{m}\left(t\right)\right)$, and the complex modulus of the resting pseudophase ${G_{r}}^{*}\left(\omega \right)=F\left({\tilde{\sigma }}_{r}\left(t\right)\right)/F\left({\tilde{\varepsilon }}_{r}\left(t\right)\right)$ for both cell configurations (where *F*[∘] is the Fourier operator for the stresses and strains of the cell surface and the pseudophases and *ω* is the angular velocity equal to *ω* = 2*π*/*t*). The cell surface complex modulus for the mixed coupling mode under equilibrium conditions described by *ϕ*_Pm_^eq^, *ϕ*_Pr_^eq^, *ϕ*_Sm_^eq^, and *ϕ*_Sr_^eq^ can be expressed from equations (13), (14), (15), and (16) as

(17) $$\begin{array}{c} \frac{G^{*}\left(\omega \right)}{{G_{r}}^{*}\left(\omega \right)}={\phi_{\text{Pm}}}^{\text{eq}}\frac{{G_{\text{m}}}^{*}\left(\omega \right)}{{G_{\text{r}}}^{*}\left(\omega \right)}+{\phi_{\text{Pr}}}^{\text{eq}}+{\left({\phi_{\text{Sm}}}^{\text{eq}}+{\phi_{\text{Sr}}}^{\text{eq}}\right)}^{2}\frac{1}{\left({\phi_{\text{Sm}}}^{\text{eq}}/\left({G_{\text{m}}}^{*}\left(\omega \right)/{G_{\text{r}}}^{*}\left(\omega \right)\right)\right)+{\phi_{\text{Sr}}}^{\text{eq}}}. \end{array}$$

Equation (17) should satisfy following conditions: (1) if *ϕ*_Pm_^eq^ → 0 and *ϕ*_Sm_^eq^ → 0 while *ϕ*_Pr_^eq^ + *ϕ*_Sr_^eq^ = 1, then *G*^∗^(*ω*)/*G*_r_^∗^(*ω*) → 1 and (2) if *ϕ*_Pr_^eq^ → 0 and *ϕ*_Sr_^eq^ → 0 while *ϕ*_Pm_^eq^ + *ϕ*_Sm_^eq^ = 1, then *G*^∗^(*ω*)/*G*_*r*_^∗^(*ω*) → *G*_*m*_^∗^(*ω*)/*G*_*r*_^∗^(*ω*).

Lange and Fabry reported that muscle cells can change their elastic modulus by over one order of magnitude from less than 10 kPa in a relaxed (resting) state to around 200 kPa in a fully activated (migrating) state caused by the accumulation of the contractile energy [16]. The higher modulus ratio, which corresponds to stiffer multicellular surfaces, is obtained for the higher value of the volume fraction of migrating cells in the form of monolayer sheets *ϕ*_Pm_^eq^. A higher value of *ϕ*_Pm_^eq^ could be realized for higher kinetic constants *k*_1_ and *k*_2_ relative to *k*_3_ and *k*_4_. This scenario corresponds to ordered cell rearrangement with minimal effects of mechanical perturbations caused by stress accumulation and biochemical perturbations.

Characteristic equilibrium states for cellular configurations obtained during cell surface rearrangement after uniaxial compression are estimated by equation (7), and the corresponding surface stiffness could be predicted by equation (17). Some characteristic equilibrium states for corresponding low, medium, and large mechanical and biochemical perturbations (i.e., the quantifications of various types of uncorrelated motility) are discussed: (1) *ϕ*_r_^eq^ → 0, while *ϕ*_Sm_^eq^ + *ϕ*_Pm_^eq^ → 1, (2) *ϕ*_r_^eq^ → 1, while *ϕ*_Sm_^eq^ + *ϕ*_Pm_^eq^ → 0, (3) *ϕ*_Sm_^eq^≻≻*ϕ*_Pm_^eq^, (4) *ϕ*_Sm_^eq^≺≺*ϕ*_Pm_^eq^, and (5) *ϕ*_r_^eq^ = *ϕ*_Sm_^eq^ ≈ *ϕ*_Pm_^eq^. We presented 5 characteristic cases in Table 1.

Various 2D and 3D experimental systems will be discussed in the context of postulated characteristic cases presented as the result of our modeling considerations.

## 4. Results and Discussion

The influence of uncorrelated motility to collective cell migration is estimated theoretically on two model systems: (1) cell aggregate rounding after uniaxial compression and (2) cell aggregate micropipette aspiration based on the proposed model. Both systems satisfy the following conditions: (1) cell long-time rearrangement is influenced by external stress, locally or globally, (2) it occurs via collective cell migration within the aggregate 3D surface region or its part, and (3) the rearrangement is driven by tissue surface tension and could be described by the Young-Laplace law [6]. The model could be also applied to 2D epithelium by considering the interrelations between surface fractions of migrating and resting cells rather than volume fractions.

Although cell migration has long been studied, the manner in which the stochastic effects influence cell rearrangement within the precisely controlled process of development remains largely unknown [37]. Stochastic effects as the product of mechanical and biochemical perturbations lead to uncorrelated motility. The generation of these perturbations is difficult to measure experimentally. Only the result of these stochastic effects could be measurable in the context of (1) configuration changes of migrating cells, (2) velocity distribution, and (3) stiffness distribution. Uncorrelated motility accounts for internal and external effects. Internal effects are caused by stress accumulation within migrating cell groups during their intercalation through dense cellular environment [23]. External effects represent the consequence of the collision of velocity fronts which is significant even in 2D [27].

Uncorrelated motility induces changes of volume fraction and configuration of migrating cells. Consequently, it represents the main cause of change of the relaxation rate of the aggregate shape during its rounding from cycle to cycle [13]. The average duration of the single cycle was 1-2 h. Every relaxation rate could be related to the various scenarios of cell migration. Three scenarios were considered base on experimental data by Mombach et al. [5]: (1) most of the cells migrate all the time which corresponds to the highest value of the relaxation rate *k*_m_, (2) some cell groups migrate while the others (at the same time) stay in the resting state which corresponds to the median value of the relaxation rate *k*_t_, and (3) most of the cells stay in the resting state which corresponds to the relaxation rate *k*_r_ ≈ 0. These three scenarios will be discussed based on the proposed model in the form of simulation.

**Figure 2:**
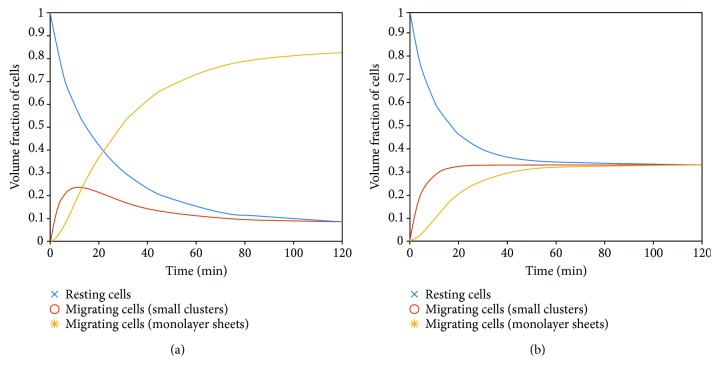
(a) Configuration state of migrating cells as a function of time for the first scenario accomplished for *k*_3_≻≻*k*_4_ and *k*_1_ = 0.067min^−1^, *k*_2_ = 0.067min^−1^, *k*_3_ = 0.1min^−1^, *k*_4_ = 0.01min^−1^. (b) Configuration state of migrating cells as a function of time for the second scenario accomplished for *k*_1_ ≈ *k*_2_ ≈ *k*_3_ ≈ *k*_4_ = 0.067min^−1^.

For this simulation, the rate constant *k*_1_ is supposed to be *k*_1_ = *t*_p_^−1^ (where *t*_p_ is the cell persistence time supposed to be equal to *t*_p_ ≈ 15min as reported by McCann et al. [25] and equal to *k*_1_ = 0.067min^−1^). The first scenario corresponds to the large volume fraction of migrating cells which could be accomplished under low or medium mechanical perturbations and low biochemical perturbations. Characteristic case 4 presented in Table 1 could be the most suitable. For this condition, the equilibrium configuration is *ϕ*_Sm_^eq^≺≺*ϕ*_Pm_^eq^, while *ϕ*_Pr_^eq^ ≈ *ϕ*_r_^eq^. Corresponding surface stiffness is quantified as *G*^∗^(*ω*)/*G*_*r*_^∗^(*ω*) = *ϕ*_Pm_^eq^*G*_m_^∗^(*ω*)/*G*_*r*_^∗^(*ω*). The simulation of the first scenario of cell migration is related to the volume fraction of resting cells, volume fraction of migrating cells in the form of small clusters, and volume fraction of migrating cells in the form of monolayer sheets to the following conditions *k*_3_≻≻*k*_4_, *k*_3_ ≥ *k*_1_, and *k*_1_ ≥ *k*_2_. Consequently, the proposed values of kinetic constants are *k*_1_ = 0.067min^−1^, *k*_2_ = 0.067min^−1^, *k*_3_ = 0.1min^−1^, and *k*_4_ = 0.01min^−1^. The result of simulation for the first scenario is shown in Figure 2(a).

A second scenario in which some cell groups migrate while the others (at the same time) stay in the resting state corresponds to medium mechanical and biochemical perturbations. This configuration of migrating cells is more disordered than the one proposed for the first scenario. For this condition, case 5 should be the most suitable (Table 1). For this condition, the equilibrium configuration is *ϕ*_r_^eq^ = *ϕ*_Sm_^eq^ ≈ *ϕ*_Pm_^eq^. The corresponding surface stiffness is quantified as *G*^∗^(*ω*)/*G*_r_^∗^(*ω*) = 0.33*G*_m_^∗^(*ω*)/*G*_r_^∗^(*ω*). The simulation of this scenario is described by the conditions *k*_1_ ≈ *k*_2_ ≈ *k*_3_ ≈ *k*_4_ = 0.067min^−1^. The result of simulation for first scenario is shown in Figure 2(b).

The third scenario corresponds to case 2 (Table 1) accomplished for large mechanical and biochemical perturbations, such that *ϕ*_r_^eq^ → 1. For this condition, the surface stiffness is *G*^∗^(*ω*)/*G*_r_^∗^(*ω*) → 1.

Instead of cell aggregate uniaxial compression between parallel plates, aggregate micropipette aspiration is another widely used experimental system for considering a collective cell migration and its influence to viscoelasticity at the supracellular level. Experimental data by Guevorkian et al. [7] is considered based on the proposed model. *N* cells from the aggregate surface part is mechanically perturbed under external pressure and inserted into the pipette. This length depends on the magnitude of applied pressure. Lower pressure of 500 Pa induces activation of perturbed cells and their entrance into the pipette via collective cell migration during 120 min. However, under higher pressure, one part of perturbed cells is instantaneously inserted into the pipette. These cells undergo collective cell migration within the pipette while the other part of already perturbed cells enters into the pipette. Guevorkian et al. detected short-time contractions with the period of 3-4 minutes and long-time contractions with the period of 10-17 minutes [7]. Short-time contractions correspond to single-cell contractions, while the long-time contractions account for collective phenomena of cell rearrangement. The period of long-time contractions corresponds to the order of magnitude of the cell persistence time. Consequently, the persistence time could be *t*_p_ ≈ 15min, while the corresponding value of the kinetic constant could be *k*_1_ = 0.067min^−1^ (the same as was supposed for the previous system). We are interested here in the response of perturbed cells under pressure of 500 Pa by collective cell migration without significant deformations of single cells that are observed under higher pressure. Corresponding cell entrance into the pipette considered by Guevorkian et al. can be estimated within two time regimes: (1) initial regime for *t* ∈ [0, 50min] and (2) final regime for *t* ∈ [50, 120min] [7]. The long-time contractions are more intensive in the final regime compared with the initial regime pointed to the disordered configuration of migrating cells. Both regimes should be characterized by the large volume fraction of migrating cells within the pipette. This assumption is in accordance with the phenomenon description by Guevorkian et al. [7]. Consequently, the initial regime can be identified as characteristic case 4 (Table 1) suitable for larger volume fractions of migrating cells and more ordered configurations. We supposed that *k*_1_ ≈ *k*_2_. This assumption can be suitable for lower pressure~ 500 Pa. For higher pressures, kinetic constant *k*_2_ should be *k*_2_≺*k*_1_. Case 4 also introduces the condition that *k*_3_≻≻*k*_4_. Consequently, the values of the kinetic constants could be similar as the ones presented in Figure 2(a), (*k*_1_ = 0.067min^−1^, *k*_2_ = 0.067min^−1^, *k*_3_ = 0.1min^−1^, *k*_4_ = 0.01min^−1^). The final regime corresponds to the more disordered configuration of migrating cells. This regime could be identified as case 5 (Table 1) for the conditions *k*_1_ ≈ *k*_2_ ≈ *k*_3_ ≈ *k*_4_ = 0.067min^−1^. The result of this simulation is already presented in Figure 2(b).

Collective cell migration during wound healing could be treated as 2D dynamics. Mikami et al. [20] and Chen et al. [30] considered this phenomenon experimentally and theoretically. They described the cell epithelial monolayer as the highly ordered configuration of migrating cells. This configuration of migrating cells leads to a stiffer surface than the disordered one. In the context of our model, this dynamic could be described by case 1 (Table 1). The long-time cell rearrangement of 2D systems could be more ordered compared with 3D systems.

Bearing in mind that mathematical models represent only the simplified description of this complex stochastic phenomenon, we point to the importance of understanding of the cause-consequence relation between (1) mechanical and biochemical perturbations generated during collective cell migration, (2) configuration changes of migrating cells, and (3) long-time viscoelasticity of multicellular surfaces.

## 5. Conclusions

A significant difference in cell stiffness between migrating and resting cell groups indicates that volume fraction of migrating cells and their distribution could influence long-time viscoelasticity of multicellular surfaces. This aspect of cell surface rearrangement could be treated by the analogy with physics in the form of two phase blends consisting of migrating and resting cell pseudophases. Migrating cells could form three different configurations: (1) monolayer sheets (lamellar structure), (2) small clusters, and (3) and various combinations of these two types. The lamellar structure of pseudoblends corresponds to parallel mode coupling, while the highly dispersed system in forms of small clusters points to series mode coupling. Mixed mode coupling is between them and accounts for both configuration types.

We proposed the two-step Eyring model for indicating main mechanical and biochemical factors which influence configurations of migrating cells as follows: (1) accumulation of mechanical stress which leads to resting-to-migrating cell state transition and vice versa and (2) cohesiveness difference between migrating and resting pseudophases which leads to configuration changes of migrating cells from small cell clusters to monolayer sheets and vice versa. Both factors induce internal response of multicellular surfaces in the context of internal rearrangement of stress, cell signaling, and the generation of mechanical and biochemical perturbations. These perturbations are the product of uncorrelated motility. Uncorrelated motility causes a decrease of the volume fraction of migrating cells and weakening of the multicellular surface. Cell rearrangement and its impact on viscoelasticity of multicellular surfaces were elaborated on two model systems: (1) cell aggregate rounding after uniaxial compression and (2) cell aggregate micropipette aspiration under pressure. This model could be also applied for considering the long-time cell rearrangement under various types of stress.

These complex phenomena provide motivation for future experiments which relate cell configurations with the rheological behavior of the multicellular surface and clarify the impact of uncorrelated motility on cell rearrangement. Experiments of the cell long-time rearrangement under stress should be combined with additional rheological experiments to characterize the surface stiffness distribution and the rate of its changes.

## Figures and Tables

**Table 1:** Characteristic case description for equilibrium configuration of migrating cells.

Case	Kinetic constants	Equilibrium volume fractions of cells	Complex modulus as a measure of the surface stiffness	Mechanical and biochemical perturbations
1	*k*_1_≻≻*k*_2_ *k*_3_≻≻*k*_4_ *k*_3_ ≈ *k*_1_	*ϕ*_r_^eq^ → 0	$\frac{G^{*}\left(\omega \right)}{{G_{\text{r}}}^{*}\left(\omega \right)}\to \frac{{G_{\text{m}}}^{*}\left(\omega \right)}{{G_{\text{r}}}^{*}\left(\omega \right)}$	Low mechanical perturbations
2	*k*_1_≺≺*k*_2_ *k*_3_≺≺*k*_4_ *k*_4_ ≈ *k*_2_	*ϕ*_r_^eq^ → 1	$\frac{G^{*}\left(\omega \right)}{{G_{\text{r}}}^{*}\left(\omega \right)}\to 1$	Large mechanical perturbations and large biochemical perturbations
3	*k*_3_≺≺*k*_4_ *k*_4_ ≥ *k*_1_ *k*_1_ ≥ *k*_2_	*ϕ*_Sm_^eq^≻≻*ϕ*_Pm_^eq^ *ϕ*_Sr_^eq^ ≈ *ϕ*_r_^eq^	$\frac{G^{*}\left(\omega \right)}{{G_{\text{r}}}^{*}\left(\omega \right)}\approx \frac{{\left({\phi_{\text{Sm}}}^{\text{eq}}+{\phi_{\text{Sr}}}^{\text{eq}}\right)}^{2}}{\phi_{\text{Sr}}^{\text{eq}}}$	Low or medium mechanical perturbations and large biochemical perturbations
4	*k*_3_≻≻*k*_4_ *k*_3_ ≥ *k*_1_ *k*_1_ ≥ *k*_2_	*ϕ*_Sm_^eq^≺≺*ϕ*_Pm_^eq^ *ϕ*_Pr_^eq^ ≈ *ϕ*_r_^eq^	$\frac{G^{*}\left(\omega \right)}{{G_{\text{r}}}^{*}\left(\omega \right)}={\phi_{\text{Pm}}}^{\text{eq}}\frac{{G_{\text{m}}}^{*}\left(\omega \right)}{{G_{\text{r}}}^{*}\left(\omega \right)}$ where *ϕ*_Pm_^eq^≻0.33	Low or medium mechanical perturbations and low biochemical perturbations
5	*k*_1_ ≈ *k*_2_ ≈ *k*_3_ ≈ *k*_4_	*ϕ*_r_^eq^ = *ϕ*_Sm_^eq^ ≈ *ϕ*_Pm_^eq^	$\frac{G^{*}\left(\omega \right)}{{G_{\text{r}}}^{*}\left(\omega \right)}=0.33\frac{{G_{\text{m}}}^{*}\left(\omega \right)}{{G_{\text{r}}}^{*}\left(\omega \right)}$	Medium mechanical and biochemical perturbations

## Data Availability

This is theoretical paper without original data. We used data from the literature.

## Appendix

### A. Two-Step Model Formulation

Cell surface rearrangement during aggregate rounding after compression satisfied the condition that the volume of the perturbed surface region is constant and equal to *V*_surf_ = *A*_surf_(*t*) *l*_surf_(*t*) (where *A*_surf_(*t*) is the aggregate surface and *l*_surf_(*t*) is the thickness of the aggregate surface region). The number of cells in the surface region is constant, i.e., *N* = const. Consequently, our multicellular surface systems behave as canonical ensemble. This is the necessary condition for applying our two-phase model. Model equation (5) in the context of the volume fractions was formulated from the following equations:

(A.1) $$\begin{array}{c} N=\text{const},\\ \frac{{dN}_{\text{r}}\left(t\right)}{dt}=-k_{1}N_{\text{r}}\left(t\right)+k_{2}N_{\text{Sm}}\left(t\right),\\ \frac{{dN}_{\text{Sm}}\left(t\right)}{dt}=k_{1}N_{\text{r}}\left(t\right)-\left(k_{2}+k_{3}\right)N_{\text{Sm}}\left(t\right)+k_{4}N_{\text{Pm}}\left(t\right),\\ \frac{{dN}_{\text{Pm}}\left(t\right)}{dt}=k_{3}N_{\text{Sm}}\left(t\right)-k_{4}N_{\text{Pm}}\left(t\right), \end{array}$$

where *N*_r_(*t*) is the number of resting cells, *N*_Sm_ is the number of migrating cells in the form of small clusters, and *N*_Pm_ is the number of migrating cells in the form of monolayer sheets.

### B. Formulation of the Mechanical Barrier

During the aggregate compression, cells are in a passive (resting) state. Part of surface cells becomes active since they are losing the equilibrium number of adhesion complexes. It is in accordance with the fact that the aggregate surface *A* increases during the time period of compression. We suppose that compression cannot induce changes of the single-cell volume. External stress relates to the internal stress (force moments) of the cells. Consequently, internal stress function $\Delta {\tilde{\Pi }}_{\text{eff}}$ is equilibrated with the external stress ${\tilde{\sigma }}_{\mathrm{ext}}$ such that *Π*_*ij*_ = *σ*_ext*ij*_ during aggregate compression. Internal stress could be expressed as

(B.1) $$\begin{array}{c} \Delta {\tilde{\Pi }}_{\text{eff}}=\frac{1}{V^{\alpha }}\sum_{\alpha ,\beta }{\vec{f}}^{\alpha \beta }{\vec{r}}^{\alpha \beta }, \end{array}$$

where *V*^*α*^ is the volume of the single cell, ${\vec{f}}^{\alpha \beta }$ is the force acting on cell *β* from cell *α*, and ${\vec{r}}^{\alpha \beta }$ is the vector from the center of *α* to the center of *β*. The entropy of cell rearrangement could be expressed as

(B.2) $$\begin{array}{c} S=S\left(V_{\text{surface}},{\tilde{\sigma }}_{\mathrm{ext}},N\right), \end{array}$$

where *S* is the surface entropy and *V*_surface_ is the surface volume. Edwards and Grinev [38] and Edwards [39] considered the rearrangement of granular systems under external stress and introduced thermodynamic-intensive variable $\tilde{Z}$ by the analogy with temperature defined as

(B.3) $$\begin{array}{c} \frac{1}{\tilde{Z}}=\frac{\partial S}{\partial {\tilde{\sigma }}_{\text{in}}}, \end{array}$$

where ${\tilde{\sigma }}_{\text{in}}$ is the internal stress which is equilibrated with the external stress ${\tilde{\sigma }}_{\mathrm{ext}}$. At the first site, it is a quite different system from the one considered here. But, more careful examination reveals that many of their properties can be defined in the same terms for dense cell populations.

### C. Formulation of the Biochemical Barrier

The biochemical barrier for configuration changes of migrating cells (second step) is expressed as the spreading coefficient. Spreading coefficient accounts for cohesiveness of both phases and interfacial tension [40]. Consequently, the corresponding energy barrier could be expressed as

(C.1) $$\begin{array}{c} \Delta E_{\mathrm{int}}=\gamma_{\text{r}}\Delta A_{\text{m}}-\left(\gamma_{\text{m}}+\gamma_{\mathrm{int}}\right)\Delta A_{\text{m}}, \end{array}$$

where *γ*_r_ is the equilibrium surface tension of the resting cell pseudophase, *γ*_m_ is the equilibrium surface tension of the migrating cell pseudophase, *γ*_int_ is the interfacial tension, and Δ*A*_m_ is the surface change of migrating cell groups. Surface tension represents the specific measure of the surface free energy.

The effective driving force for the second step Δ*E*_eff_ (i.e., driving energy) comes from cell signaling. This biochemical driving force could be expressed as

(C.2) $$\begin{array}{c} \Delta E_{\text{eff}}=\Delta E_{\text{S}}-\Delta E_{\text{P}}, \end{array}$$

where Δ*E*_P_ is the energy perturbation and Δ*E*_S_ accounts for cell signaling which manages the cohesiveness of migrating and resting cell pseudophases near the interface expressed as

(C.3) $$\begin{array}{c} \Delta E_{\text{S}}=k_{B}T_{\text{eff}}\Delta H, \end{array}$$

where *k*_*B*_ is the Boltzmann constant and *T*_eff_ is the effective temperature expressed based on generalization of Einstein's relation [26]. Consequently, the effective temperature could be expressed as

(C.4) $$\begin{array}{c} k_{B}T_{\text{eff}}=\frac{D}{\mu^{\prime }}, \end{array}$$

where *D* is the diffusivity of migrating cells and *μ*′ is the mobility of velocity fronts. The variable Δ*H* represents Shannon information entropy which has been applied to cell signaling and expressed by [41]

(C.5) $$\begin{array}{c} \Delta H=H\left(I\right)-H\left(\frac{I}{R}\right), \end{array}$$

where *H*(*I*) can be interpreted to be the overall uncertainty about the input *S* and *H*(*I*/*R*) is the residual uncertainty about *S* after the value of the response *R* is known. Energy perturbation Δ*E*_P_ could be equal to

(C.6) $$\begin{array}{c} \Delta E_{\text{P}}\approx k_{B}T_{\text{eff}}. \end{array}$$

The perturbations of cell signaling could biochemically reduce the action of the driving energy and inhibit conformation changes of migrating cells.
